# Molecular complementarity between simple, universal molecules and ions limited phenotype space in the precursors of cells

**DOI:** 10.1186/s13062-014-0028-3

**Published:** 2014-12-04

**Authors:** Vic Norris, Rosetta N Reusch, Kazuei Igarashi, Robert Root-Bernstein

**Affiliations:** Department of Biology, University of Rouen, 76821 Mont Saint Aignan, France; Department of Microbiology and Molecular Genetics, Michigan State University, East Lansing, MI 48824 USA; Amine Pharma Research Institute, Innovation Plaza at Chiba University, 1-8-15 Inohana, Chuo-ku, Chiba 260-0856 Japan; Department of Physiology, Michigan State University, East Lansing, MI 48824 USA

**Keywords:** Origin of life, Protein kinase, Hyperstructure, Network, Cation, Polymer, Complementarity, DNA, RNA

## Abstract

**Background:**

Fundamental problems faced by the protocells and their modern descendants include how to go from one phenotypic state to another; escape from a basin of attraction in the space of phenotypes; reconcile conflicting growth and survival strategies (and thereby live on ‘the scales of equilibria’); and create a coherent, reproducible phenotype from a multitude of constituents.

**Presentation of the hypothesis:**

The solutions to these problems are likely to be found with the organic and inorganic molecules and inorganic ions that constituted protocells, which we term *SUMIs* for Simple Universal Molecules and Ions. These SUMIs probably included polyphosphate (PolyP) as a source of energy and of phosphate; poly-(R)-3-hydroxybutyrate (PHB) as a source of carbon and as a transporter in association with PolyP; polyamines as a source of nitrogen; lipids as precursors of membranes; as well as peptides, nucleic acids, and calcium. Here, we explore the hypothesis that the direct interactions between PHB, PolyP, polyamines and lipids – modulated by calcium – played a central role in solving the fundamental problems faced by early and modern cells.

**Testing the hypothesis:**

We review evidence that SUMIs (1) were abundant and available to protocells; (2) are widespread in modern cells; (3) interact with one another and other cellular constituents to create structures with new functions surprisingly similar to those of proteins and RNA; (4) are essential to creating coherent phenotypes in modern bacteria. SUMIs are therefore natural candidates for reducing the immensity of phenotype space and making the transition from a “primordial soup” to living cells.

**Implications of the hypothesis:**

We discuss the relevance of the SUMIs and their interactions to the ideas of molecular complementarity, composomes (molecular aggregates with hereditary properties based on molecular complementarity), and a prebiotic ecology of co-evolving populations of composomes. In particular, we propose that SUMIs might limit the initial phenotype space of composomes in a coherent way. As examples, we propose that acidocalcisomes arose from interactions and self-selection among SUMIs and that the phosphorylation of proteins in modern cells had its origin in the covalent modification of proteins by PHB.

**Reviewers:**

This article was reviewed by Doron Lancet and Kepa Ruiz-Mirazo.

## Background

The problem of how life might be created on Earth was solved somehow during the evolution of the distant ancestors of modern cells. One approach to rediscovering this solution is therefore to interrogate modern cells. Unfortunately, over the course of billions of years, they may have forgotten this solution. A different approach is to reason that certain problems that continue to confront modern cells also confronted their ancestors. These problems are interesting because they are ongoing and the solutions to them may be accessible. Hence, an Occam’s Razor approach in which Life’s fundamental problems are the same everywhere has the advantage that modern cells can usefully be interrogated about their solutions to these problems which may then be transposed to the context of the origins of life.

The first of these fundamental problems is how to generate reproducible, coherent phenotypes from a large number of effectively different molecules. In modern cells, this number runs into thousands - if not millions –if genes and other nucleic acid sequences are considered as separate entities, if post-translational modifications are taken into account and if the small molecules of metabolism are included. The combination of the activities of these molecules generates the phenotype on which natural selection acts. Since there would appear to be an almost unlimited number of such combinations, there should be an almost unlimited number of phenotypes. Therefore, the problem for modern cells is to reduce this number so as to allow the generation of phenotypes that can not only be repeated [1] but also be coherent with respect to their environments and their histories [2]. One, partial solution adopted by cells is to organise the phenotype not at the level of a myriad molecules or macromolecules but at the higher level of a small number of hyperstructures, which are spatially extended assemblies of molecules and macromolecules that have one or more functions within the cell [3]. Such hyperstructures can command signaling molecules and macromolecules, and can perform structural and metabolic roles. The formation of hyperstructures depends to some extent on molecular complementarity. Molecular complementarity occurs when the shapes of molecules or macromolecules fit one another such that physical, non-covalent interactions result in their associating reversibly with one another [4,5]. Molecular complementarity underlies functions such as information storage and translation, enzymatic reactions, structural self-organization and protection of molecules from degradative processes. The upshot of all this is that probably less than a hundred different hyperstructures, created in part by molecular complementarity, determine the phenotype of the bacterium. This means that the number of hyperstructures is orders of magnitude less than the number of macromolecules so the number of phenotypes resulting from combinations of hyperstructures is much smaller than the number resulting from combinations of macromolecules. In other words, phenotype space is dramatically reduced but remains huge.

A second, fundamental problem is how to generate phenotypes that can satisfy the incompatible requirements of survival and growth. This is the problem of ‘Life on the scales’ whereby cells are damned if they simply grow (since they risk being destroyed if conditions turn bad) and damned if they eschew growth, for example, to sporulate (since they risk being out-competed by other, growing, cells if conditions remain good) [6]. To simplify it, at one extreme, survival requires a cell that approaches an equilibrium state in which it is relatively static and robust but does not grow (with interactions between cellular constituents that are strong and stable) whilst, at the other extreme, growth requires a cell in a non-equilibrium state in which it is highly dynamic and metabolically active but risks death (with interactions between cellular constituents that are weak and unstable). Moreover, cells must be able to go between these states. One of the solutions adopted by modern bacteria is to have a cell cycle that gives daughter cells with different phenotypes as evidenced by the division of *Caulobacter crescentus* into stalked and swarmer cells each of which can generate the other [7]. In the hyperstructure hypothesis, these different phenotypes are conferred by different combinations of equilibrium (technically, quasi-equilibrium) and non-equilibrium hyperstructures [3].

In our unifying approach, the above fundamental problems also confronted life at some stage during its origins. These problems would have arisen early on if life began as a prebiotic ecology of astronomical numbers of combinations of interacting molecules abiotically created and destroyed in a wide range of environments [8]. In this scenario, the solution again lay in molecular assemblies and molecular complementarity: molecules were abiotically created and destroyed but a subset of molecules was preserved because their complementarity led to associations between them that protected them from degradation; these interacting molecules then accumulated in the form of molecular and macromolecular assemblies or composomes – the ancestors of modern hyperstructures – which possessed new properties and which exhibited compositional inheritance [9]. For example, the synthesis of linear polymers including oligonucleotides and peptides was catalysed at interfaces between and on surfaces within the composomes [10]. These composomes evolved together as a population exchanging their contents via fission-fusion processes [11], with selection acting on composomal species that varied in properties and functions (derived from their equilibrium and non-equilibrium characteristics) to eventually yield the first cells [8] in which metabolism and replication were brought together [12].

Although the current scenario of the prebiotic ecology offers solutions to fundamental problems, these solutions are incomplete. How was phenotype space sufficiently constrained by composomes to have enabled natural selection to act effectively? How did composomes or collections of composomes go from an equilibrium state to a non-equilibrium one and back again? How exactly was energy generated and catalysis achieved? Addressing these questions by invoking peptides and oligonucleotides would be understandable. There are, however, other molecules that merit consideration. Not so long ago, the late Arthur Kornberg chided the scientific community for dismissing polyphosphate (PolyP) and its metabolism as a mere “molecular fossil” [13]. In modern cells, PolyP is implicated in quorum sensing, biofilm formation, motility, virulence, sporulation phosphate storage and energy metabolism [14]. PolyP is not alone in receiving insufficient attention. Short chain poly-(R)-3-hydroxybutyrate (PHB) can form ion and DNA uptake channels (with PolyP) and pumps [15,16] and may directly modulate interactions between proteins, nucleic acids and membranes [17,18]; moreover, PHB can act as a carbon store [19]. Polyamines bind to nucleic acids and proteins, decrease membrane permeability, may help cells survive abiotic stresses [20,21]; polyamines may also act as nitrogen stores. These molecules along with other molecules such as lipids, and inorganic ions constitute the “molecular paleontology” which exists in modern cells and which offers important clues about their evolution [4,8,22]. We term these molecules SUMIs, standing for Simple, Universal Molecules and inorganic Ions.

SUMIs play a major role in modern hyperstructures. In the case of equilibrium hyperstructures, acidocalcisomes are one of the many spatially extended, intracellular assemblies of molecules that are believed to exist in every living species [23,24]. They are rich in calcium, pyrophosphate and PolyP and perform some of the most essential functions of cellular metabolism including osmotic regulation, calcium storage and regulation, and PolyP metabolism [25]. In the case of non-equilibrium hyperstructures, compartments in which ribosomes are assembled go from the nucleolus in eukaryotes to ribosome foci in prokaryotes [26]; the ribosome foci are part of the large class of hyperstructures in which transcription and translation are coupled and, significantly, several of the proteins in these hyperstructures are modified by the covalent addition of PHB [17]. The widespread distribution of acidocalcisomes and ribosomal hyperstructures is consistent with their persistence across evolutionary time extending as far back as the composomes.

In modern cells, SUMIs such as PolyP, PHB and polyamines are involved in both growth and survival and hence are well-placed to mediate transitions between the two states. Moreover, these SUMIs are also involved in energy metabolism. Their simple, essential nature and their universality therefore it likely they were major actors in the prebiotic ecology. Here we reason that if in our beginnings we have our end, then from that end we should be able to see our beginnings, We are therefore led to propose that the SUMIs solved fundamental problems in the origins of life. We review briefly the literature for modern cells on PolyP, PHB and polyamines and on their interactions with lipids and with calcium, focusing on these SUMIs rather than on the intensely studied peptides and oligonucleotides, on oligosaccharides, and on the other inorganic ions (all of which are also SUMIs). We discuss the properties of these SUMIs with respect to concepts such as molecular complementarity, hyperstructures, and Life on the Scales. As an example of the unifying value of our proposal, we suggest that interaction between the SUMIs lies at the root of protein phosphorylation in modern cells.

## Presentation of the hypothesis

The primary hypothesis that we present here is that composomes and their functions evolved by means of a series of SUMIs that endowed prebiotic ecologies with unprecedented ways: to control ion fluxes and osmotic pressure gradients; to store carbon, nitrogen and phosphate, and to store and release energy; to select interacting molecules; to catalyze reactions and to generate polymers. More specifically, we propose that (1) a limited number of SUMIs constituted the first composomes, (2) the interactions between SUMIs determined the behavior of composomes, (3) these interactions constituted a system of global regulation based on the division of composomes that limited phenotype space to the two large attractors of growth and survival and to the transitions between them, and (4) these roles for SUMIs can still be discerned in modern cells in their universal utilization of ribosome microcompartments and acidocalcisomes to regulate key cellular processes.

In our hypothesis, abiotically created lipids, amino acids and nucleotides entered a composome that contained PolyP and the other SUMIs. The PolyP and PBH formed a granule with a semi-regular surface on which amino acids and nucleotides, for example, were concentrated, aligned and oriented such that polymerization occurred. Depending on their length and sequence, these polymers had different probabilities of dissociating from the granule. The reactions that generated these polymers did not reach equilibrium because many of these polymeric and other products were removed from the ‘reaction chamber’ of the composome by a process of growth, separation and division. This process resulted from multiple, concurrent processes: the growth of the SUMI-based composome due to the accretion of new material from the environment and to relatively non-specific catalysis on the granule inside the composome; the physical separation of these constituents based on their affinities for one another; division between the separated regions. The result was (1) to give a daughter, SUMI-based composome (that bore some similarity to the mother composome) and (2) to release both unbound molecules (that were therefore unprotected and degraded) and a second daughter composome in which the molecules bound with greater affinity to one another than to the SUMIs. Each daughter composome contained a different distribution of the SUMIs in terms of their ratios, lengths and structural configurations. Hence, at this early stage of evolution of the prebiotic ecology, peptides and oligonucleotides (as well as other molecules) increased in abundance but the range of these molecules was constrained because they were either bound to the SUMIs or generated by reactions catalyzed by them. In this way, the SUMIs generated composomes that were coherent in the sense that their constituents were all connected in some way with the SUMIs.

The diversity of coherent, SUMI-based composomes equipped the composomal population to exploit the opportunities and to overcome the challenges of a prebiotic environment characterized by major spatial and temporal changes on all scales. At one extreme of the population, non-equilibrium composomes contained SUMIs in configurations that selected particular subsets of oligonucleotides and peptides for low affinity interactions; these interactions were dynamic and the energy from PolyP was used in reactions that allowed growth whilst, for example, PHB facilitated interactions between oligonucleotides and peptides. At the other extreme, equilibrium composomes contained SUMIs that acted as storage molecules and that selected other subsets of oligonucleotides and peptides for high affinity interactions; these interactions resulted in static states that conferred robustness; however, certain changes in physical and chemical parameters in the environment altered the interactions inside this latter class of composomes and allowed them to become dynamic and grow. In addition, many composomes possessed lipid membranes into which PolyP-PHB complexes were incorporated to form a variety of pumps and channels that could transport not only ions but also oligonucleotides and that could be regulated by physical interaction with peptides.

We further propose that the above roles for the SUMIs within composomes in the prebiotic ecology can still be discerned for SUMIs in the equivalent of composomes, that is, hyperstructures, in modern cell populations. We therefore examine the evidence that the SUMIs play a role in the modern equivalents of composomes, namely, hyperstructures. The equivalents of the non-equilibrium types of composome in which macromolecules are synthesized are epitomized by the nucleolus in eukaryotic cells and by the “nucleolus-like” hyperstructures in prokaryotic cells as well as by the other prokaryotic hyperstructures in which transcription and translation are coupled. The equivalents of the equilibrium types of hyperstructures are epitomized by acidocalcisomes which help regulate osmolarity and pH and which are found in every major branch of life.

### Polyphosphate

PolyP is likely to have been abundant on the prebiotic earth because it can be produced readily from the dehydration of phosphate rock at high temperature. It was therefore a freely available source of energy that could have been used to activate the precursors of fatty acids, phospholipids, peptides and nucleic acids. In modern bacteria, polyphosphate kinase 1 or PPK1 is involved in functions that include quorum sensing, biofilm formation, motility and virulence whilst an exopolyphosphatase, PPX1, is involved in sporulation [14]. Defects in some of these functions may be related to a role for PolyP in the compaction of the nucleoid, which occurs in *ppk1* mutants of *Pseudomonas aeruginosa* [27]; it may therefore be significant that PolyP binds *in vitro* to the histone-like, HU proteins of *E. coli* and *P. aeruginosa* since such binding might displace these proteins from the DNA [14]. The cellular functions of PolyP may also depend on its role as an energy donor. As well as PPK1, many bacteria have PPK2, a kinase that uses PolyP to catalyze the formation of nucleoside triphosphates and, in particular, the formation of GTP from GDP. Moreover, several bacteria have an NAD kinase that can catalyze the formation of NADP using either PolyP or ATP and an enzyme that can catalyze the formation of glucose-6-phosphate from glucose using PolyP [28,29]. It has been proposed that the ancestor of glucose kinases in the hexokinase family was similar to glucomannokinase and used PolyP [30]. Another aspect of the role of PolyP in energy metabolism is in the response of cells to nutrient deprivation such as the lack of an amino acid; this deprivation can lead to a 100-fold increase in the level of PolyP which, in binding to the Lon protease, activates protein degradation (largely from inactive ribosomes) to yield a pool of amino acids that can be used to make other proteins [31]. During the nutrient starvation of *Helicobacter pylori*, PolyP binds to the principal sigma factor and, during such starvation, mutant strains defective in the interaction die, consistent with the authors’ suggestion that PolyP is a second messenger determining gene expression during stress in *H. pylori* and other pathogens [32].

PolyP has been implicated in the bacterial cell cycle. Addition of PolyP at a concentration of 0.05% led to the filamentation of *Bacillus cereus* with no septum formation but with apparently normal chromosome replication and segregation [33]. In *Caulobacter crescentus*, PolyP (and the alarmone ppGpp) inhibit the swarmer-to-stalked transition; moreover, in conditions of carbon depletion in which swarmer cells should not initiate chromosome replication, swarmer cells that are unable to make PolyP or ppGpp not only initiate chromosome replication but also cleave the replication inhibitor CtrA and develop polar stalks, consistent with PolyP being a major cell cycle regulator [34].

Finally, PolyP is believed to be central to the survival strategies of certain modern bacteria. At low temperatures, when phosphate is available but nitrogen is scarce, *Yersinia pseudotuberculosis* and *Listeria monocytogenes* store the phosphate as PolyP for future use [35]. In the cyanobacterium, *Aphanizomenon ovalisporum*, the huge increase in DNA as dormant cells are formed probably depends on phosphate provided by PolyP bodies; this increase is believed to allow long-term survival during stress and a subsequent rapid resumption of metabolism and cell division when conditions improve [36]. Similarly, *Corynebacterium glutamicum* stores PolyP when phosphate is available and the partitioning between cytosolic and granular forms of PolyP is dynamical [37].

### Poly-(R)-3-hydroxybutyrate

PHB is a linear head-to-tail homopolymer of (R)-(3-hydroxybutyric acid). In a wide variety of bacteria, PHB is often known as a polymer of high molecular mass that constitutes a carbon store in the form of crystalline cytoplasmic granules that may constitute up to 90% of the cell’s dry weight [19]. However, a short chain form of PHB with a low molecular mass (<150 residues) has been found in all prokaryotes and eukaryotes examined to date. In *E. coli*, synthesis of this PHB depends on fatty acid metabolic pathways and short fatty acid catabolism, via the expression of genes in the *atoDAEB* operon as regulated by the AtoSC system [38]. AtoS is a membrane-bound histidine kinase that undergoes auto-phosphorylation in response to acetoacetate; AtoS then phosphorylates the response regulator AtoC which in turn activates transcription of the *atoDAEB* operon to allow growth on short-chain fatty acids.

The short chain form of PHB forms a non-covalent complex with PolyP in the membranes of bacteria and mitochondria [39-41] which functions as a calcium-selective channel as shown by both reconstitution experiments using cell extracts and *in vitro* experiments using chemically synthesized constituents [15]. This complex has many of the characteristics usually attributed to proteinaceous calcium channels, namely, selectivity for divalent over monovalent ions, a high permeance to calcium, strontium and barium ions, blockage by low concentrations of transition metal cations such as lanthanum, and, remarkably, voltage-activation and voltage-dependence [42]. PolyP and PHB were also found to be components of the calcium-transporting transient receptor potential channel TRPM8, a sensor of low temperature and cooling agents in the peripheral nervous system [43].

An ion channel role for the PHB/PolyP complex extends to potassium ions. In the potassium channel of *Streptomyces lividans*, KcsA, each of the constituent four polypeptides is covalently modified by very short chain PHB (<15 residues). These PHB-modified polypeptides surround a PolyP core that attracts, binds, and conducts K^+^ in response to an electrochemical stimulus whilst the polypeptides determine the selectivity for this ion by preventing access to the PolyP chain at the extracellular side (‘selectivity filter’) and by surrounding the PolyP chain with arginines at the cytoplasmic side to discourage binding of divalent cations [44]. A role for PolyP and PHB may extend to many other proteinaceous ion channels. For example, the mammalian calcium-transporting channel TRPM8, mentioned above, which forms a complex with PolyP [43], is modified by covalent attachment of very short chain PHB [45].

PHB/PolyP complexes are also involved in the uptake of DNA from the environment. When *E. coli* is subjected to the stresses leading to competence, it produces up to 20% more PHB/PolyP complexes than when growing exponentially [17]. Divalent cations such as Mg^2+^, Ca^2+^, Mn^2+^ and Sr^2+^ may cross-bridge phosphate residues of PolyP to phosphate residues of one strand of DNA. Following a transition back to normal growth conditions, the excess complexes are withdrawn from the membrane thereby transporting the bound single-stranded DNA through the PHB channel and into the cytoplasm. In support of this hypothesis, when DNA uptake is interrupted in *E. coli,* single-stranded DNA can be found complexed to PHB [42].

PHB/PolyP complexes may also be at the origin of pumps responsible for the efflux of ions from the cell [46]. Both PHB and PolyP are present in a very common pump, the Ca^2+^-ATPase of human erythrocyte membranes [16]. The putative mechanism is that the Ca(PolyP) chain is secreted through the PHB sheath by addition of phosphate from ATP to the PolyP chain at the cytoplasmic end and degradation of PolyP at the periplasmic face by exopolyphosphatases. This is a feasible mechanism since the ionic bonds between PolyP and Ca^2+^ are very strong whilst the ion-dipole bonds between PHB and Ca^2+^ are relatively weak.

Covalent modification of proteins by PHB is widespread. In *E. coli*, most PHB is associated with the ribosomal fraction. PHB-modified proteins include: the porins, OmpA, OmpF, OmpC and OmpW; one of the principal proteins of the outer membrane, Lpp; a subunit of the F1 ATP synthase, AtpD; elongation factors EF-Tu and EF-Ts; heat shock proteins, GroEL and DnaK; ribosomal proteins RplL and S1; histone-like protein, H-NS; RNA polymerase subunit, RpoA [17,18]. It is likely that the covalent modification by PHB of proteins underlies many interactions between these proteins and other proteins or membranes or nucleic acids. One reason for this is that such modification would make the proteins less water-soluble and more lipid-soluble. Hence, the amphiphilicity and flexibility of PHB would facilitate the interaction of these proteins with nucleic acids and with membranes and, indeed, their interaction with one another as in the modern ribosome.

Like its PolyP partner, PHB is probably important for survival and for transitions between growth regimes. First, PHB can act as a carbon source to be used when growth conditions permit. Second, changes in ion levels often accompany transitions between growth and survival conditions (as mentioned above) and, here again, there is a clear role early in evolution for PHB at the heart of channels and pumps.

It is tempting to include PHB modification to many key proteins in a general role for PolyP/PHB in stress conditions and in transitions. If PHB modification is needed for the functioning of many proteins, it might be expected that mechanisms would have evolved to regulate such modification. An obvious mechanism would be the phosphorylation by protein kinases of the residues on proteins that would otherwise undergo PHB modification, coupled with the reciprocal action of phosphatases. In *E. coli* and other bacteria, phosphorylation on serine, threonine and tyrosine residues is common (in addition to the better-known phosphorylation on histidine) [47-49]. It is therefore significant that these residues are modified by PHB. For example, serine residues S163 and S167 of the sorting signal of OmpA are modified by PHB to allow OmpA to be incorporated as a narrow pore into lipid bilayers at room temperature [50]. Finally, we note that, as a phosphate donor, PolyP is often associated with PHB in proteins; early in evolution, this may have put PolyP in the right place at the right time to help modify a residue with a phosphate.

### Polyamines

The synthesis of polyamines involves the conversion of L-arginine to ornithine via arginase followed by the decarboxylation of ornithine by ornithine decarboxylase to give the diamine putrescine which is the precursor for the triamine spermidine (via spermidine synthase) and tetramine spermine (via spermine synthase) [51]. Polyamines were believed to be present in all cells in the three kingdoms until recently when a few bacterial species, such as *Staphylococcus aureus*, were found to lack the genes needed for their synthesis and, in the case of *S. aureus*, to be able to grow in their absence [52]. Polyamines like putrescine, spermidine, spermine and cadaverine are required for the growth of *E. coli* where they mainly exist in the form of polyamine–RNA complexes [53]. Spermidine and spermine can also bridge the major and minor grooves of DNA, clamping together two different molecules or two parts of the same molecule [54]. In eukaryotes, lowering polyamine levels results in the partial unwinding of DNA and the unmasking of previously buried sequences that are potential binding sites for factors regulating transcription [55]. In addition to interacting with nucleic acids and binding to ribosomes, they block porins and decrease membrane permeability, and they stimulate the synthesis of proteins encoded by the “polyamine modulon” [20]. Many of these proteins are global regulators including the sigma factors RpoS, FecI, RpoN, and related RNA polymerase omega subunit RpoZ, the adenylate cyclase Cya, the transcription factor, Cra, which senses the glycolytic flux, SpoT which catalyzes both the hydrolysis and synthesis of (p)ppGpp, the effector of the stringent response, and the nucleoid-associated proteins Fis and H-NS. Recently, it has been proposed that polyamines play an important role in the stationary phase survival of *E. coli* because they stimulate the synthesis of SpoT, which leads to increased ppGpp levels in these conditions, and of RpoZ, which is needed for is necessary for the regulation of RNA synthesis with alternative sigma factors by ppGpp [56]. Polyamine functions in bacteria also include biosynthesis of siderophores, acid resistance, free radical scavenging, and biofilm formation [57,58]. It should be noted here that, in plants, polyamines are implicated in protection against a wide variety of abiotic stresses including salt and drought stress, mineral deficiencies, chilling, wounding, heavy metals, UV, ozone and paraquat [21]. Note too that covalent modification of intracellular proteins by polyamines is one of the few modifications that add positive charges, as in the case of the stabilization of microtubules by transglutaminase-catalyzed polyamination of tubulins [59].

What is the relationship between polyamines and PHB/PolyP? AtoC is the response regulator of the AtoS-AtoC two-component system that activates *atoDAEB* to produce PHB as mentioned above. AtoC is also an antizyme responsible for the post-translational inhibition of polyamine biosynthetic enzymes [60]. Polyamines and PolyP interact directly [61]. Moreover, a *speABC* and *cadA* mutant of *E. coli,* which is deficient in polyamines, accumulates less PolyP during under conditions of amino acid starvation than the wild type whilst a *speG* mutant, which has higher levels of polyamines, accumulates more PolyP; given the increased stability *in vitro* conferred by polyamines on PolyP granules, the authors concluded that polyamines interact with PolyP and affect PolyP accumulation [62].

### Interactions with lipids

Lipids have long been implicated in the origins of life [11,63-65]. Their interactions with the other constituents of cells, such as ribosomes, have been shown to result in the accumulation of these constituents within liposomes [66]. In modern bacteria, interactions between phospholipids and the nascent peptides generated *via* coupling between transcription, translation and protein insertion into membrane are considered to structure the membrane into domains that help determine the phenotype [67]. Such interactions are a prime example of the importance of molecular complementarity.

Polyamines also interact with lipids. Interaction between polyamines and acidic phospholipids is an ionic one such that the strongest interaction is between the polyamine with the highest positive charges and the phospholipid with the highest content of negative groups [68]. By bridging proteins and lipids and by shielding the surface charges, interaction with polyamines may reduce the repulsive forces between negatively charged membrane components [69]. Molecular complementarity is based on interactions between molecules that protect them from degradation and lead to their accumulation. In line with this, exogenous polyamines help protoplasts and spheroplasts resist osmotic shocks [70-72]; intracellular physiological concentrations of spermine and spermidine increase the mechanical stability of resealed erythrocyte ghosts [73]; importantly, the binding of polyamines to the polar head-groups of the membranes helps protect them from lipid peroxidation [74,75]. It has been suggested that polyamine binding to the acidic groups of phospholipids in membranes could lead to the clustering (and possibly phase-separation) of these phospholipids and even a bridging between acidic phospholipid domains of closely apposed membranes [69]. The latter possibility would give a major role to polyamines in the fission-fusion process between composomes in the prebiotic ecology scenario [11]. Finally, in this context, the maintenance of bilayer integrity must have been of fundamental importance early in the origins of life. *in vitro*, spermine stabilized a phospholipid membrane whilst putrescine and spermidine destabilized it [76]: *in vivo*, intracellular polyamines affected the flip-flop of phospholipids across the plasma membrane during apoptosis [77].

### Interactions with calcium

Protocells would have had to cope with problems such as the solubility of phosphate posed by huge amounts of extracellular calcium and it may be that, in overcoming these problems, calcium became involved in many different processes as evidenced in modern bacteria [78,79]. Moreover, calcium can interact with several of the simple constituents of modern cells that were likely constituents of protocells. For example, PolyP displays a strong preference for calcium over other physiological cations. Its preference for divalent over monovalent cations may be attributed to their higher binding energies, and the preference for calcium over magnesium ions to a lower hydration energy [80]. In the quest for solutions to the fundamental problem of generating coherent phenotypes, it could thus be argued that calcium was well-placed right from the start to play a unifying role by interacting directly or indirectly with PolyP, PHB, polyamines and phospholipids. This role continues in modern cells where calcium helps coordinate membrane and cytoskeletal structures, enzymic activity, and kinases and phosphatases.

One, unifying, role for calcium would be in helping provide a coherent response to stresses. A related role for calcium would be in the transitions between growth and survival strategies (where it might act as a kind of reset button to help cells escape from one state and progress to the next) [81]. Calcium would therefore serve as one of the factors involved in differentiation required for *life on the scales* [6]. Consistent with this, in bacteria: (1) calcium is essential for heterocyst formation [82] and for sporulation 69, (2) calcium affects growth rate via ATP levels [83] thereby putting it in a position to generate metabolic heterogeneity within a population [6], (3) calcium is involved in biofilm formation and dispersal [84-86], (4) calcium increases the expression of genes or the functioning of proteins involved in chemotaxis, swarming, virulence and motility [87-89], (5) calcium is implicated in the response to nitrogen starvation [90] and to environmental pollutants [91].

It might be argued that polyamines are the cell-made version of inorganic ions such as calcium. Both can condense as counterions onto charged membranes and filaments *in vitro*, where they can diffuse freely [92-94], and it is conceivable that such condensation also occurs *in vivo* [95,96]. Polyamines do differ significantly from ions like calcium because of their polycationic structure with positive charges distributed at fixed positions along a flexible carbon chain, thereby allowing polyamines to have different interactions from those of the metal cations [69]. Cells may have rapidly evolved systems of regulation that exploit these differences and that allow coordination of the multiple functions of the primordial molecules. Spermine and spermidine play an important role in the regulation of calcium transport in mitochondria (for references see [69]). This raises the question of the reciprocal relationship - does calcium affect polyamine levels? There is circumstantial evidence that it might. High extracellular calcium maximizes PHB accumulation via AtoS-AtoC [97] – and AtoC is the antizyme which inhibits the synthesis of polyamines [60]. Moreover, calcium channel blockers, which alter PHB levels and distribution (so as to alter, presumably, calcium influx through PHB/PolyP channels) inhibit signal transduction through AtoS-AtoC [98].

Calcium is implicated in PolyP metabolism. Acidocalcisomes, for example, are organelles that are rich in calcium, pyrophosphate and PolyP, and that contain pumps, antiporters, and channels as well as enzymes involved in the synthesis and degradation of pyrophosphate and PolyP [25]. Acidocalcisomes and the related volutin granules of PolyP are conserved across the phyla [23,24] and are thought by some to be the first, calcium-containing, acidic compartment [25]. As mentioned above, PolyP granules may interact with polyamines (note that even if those isolated from *E. coli* did not contain calcium the *phoU* mutant studied had 1000-fold higher levels of PolyP than the wild type [62]). The functions attributed to acidocalcisomes include the regulation of osmolarity, intracellular pH, and calcium levels as well as PolyP metabolism, the storage of cations and phosphorus, and the response to light [23]. In mitochondria, decreasing the level of PolyP increases the capacity of mitochondria to accumulate calcium as well as increasing NADH levels and decreasing the inner membrane potential [99].

Calcium can interact with anionic lipids to form domains and to drive membrane domain dynamics [100-102], as may also be the case for polyamines (see above). The formation of membrane domains of anionic phospholipids is intimately linked with the timing and positioning of cell division [67,103,104]. This raises the question of whether calcium plays a role, via membrane dynamics, in cell division. It may therefore be significant that addition of calcium (or magnesium) reverses the inhibition by exogeneous PolyP of cell division and even promotes minicell formation [33] since this might be attributed not only to the effect of calcium on polymerization of the key protein in bacterial division, the tubulin-like FtsZ [105], but also to the effect of calcium on membranes containing anionic phospholipids. One model system that might reveal such effects are bacterial L-forms, which are bacteria that grow without their peptidoglycan walls and which can usefully be considered as a throwback to the first cells [106,107]. An L-form of *E. coli* was found to have a five-fold lower level of FtsZ than its parent [108] whilst an engineered L-form of *Bacillus subtilis* manages to divide without FtsZ at all [109]. If the division of L-forms represents a return to the putative, membrane domain-based, division of protocells, it would also be significant that division in an *E. coli* L-form depends on calcium [108].

Extracellular calcium has been found to have a surprising effect on the growth rate of *E. coli* with a 10 mM concentration resulting in a 10% reduction in generation time [110]. What might be the mechanism? This calcium concentration also led to a 30% increase in the concentration of ATP [110]. Given that ATP can be generated from PolyP and that both calcium influx and efflux can be generated by PHB/PolyP complexes in the membrane, it might be imagined that these SUMIs were responsible. That said, deletion of the genes coding for AtoA (likely to be involved in PHB synthesis) and for PPK (involved in PHB synthesis) did not result in defective calcium efflux [110]. Another mechanism might comprise calcium condensing onto DNA, as mentioned above [92] and, significantly, calcium binds to DNA gyrase to result, it is proposed, in relaxation [111]. There may, of course, be alternative or complementary explanations involving, for example, calcium effects on the ATP synthase [112].

### From composome to hyperstructure (and back again)

At a very early stage of the evolution of the prebiotic ecology, equilibrium composomes could have exploited the free energy associated with the attraction of peptides to the boundaries of lipid domains [113] to drive polymerization. Such boundaries can concentrate, align and orientate monomers, reduce hydrolysis and increase condensation. Indeed, a model of abiotic catalysis shows the free energy change from the redistribution of peptides within domains is sufficient to drive the formation of the peptide bond; the resulting polymerization is significant and the equilibrium distribution of polymer lengths is shifted towards longer peptides. This model is supported by the observation of shifts in equilibria in reactions in organic solvents [114,115] and by the 10^11^ increase in reaction rates due to orientation effects [116].

In our hypothesis, boundaries or interfaces between combinations of SUMIs and other molecules abounded in the equilibrium composomes which dominated early prebiotic evolution. Catalysis on these interfaces resulted from the dynamic effects on the composomes of changes in the physical and chemical nature of their environment, and division to generate a network on interacting polymers. This process was guided by the molecular complementarity between the SUMIs and between the monomers and polymers with the SUMIs. Is there evidence for the existence of equilibrium composomes in the modern world of hyperstructures?

The equilibrium type of hyperstructure is well-represented by the acidocalcisome (see above). Is it reasonable to hypothesize that acidocalcisomes are the vestiges of SUMI-based composomes essential to the emergence of life? More specifically, are acidocalcisomes the descendants of composomes that were able to accumulate phosphates and store energy while regulating calcium and other ion concentrations and fluxes thereby surviving environmental stresses? Could the molecular complementarity between SUMIs involved in acidocalcisome metabolism have resulted in the formation of self-organizing composomes in which integrative interactions between SUMIs (in the appropriate conditions) converted energy into biologically useful forms such as polymerization? Universality and conservation are criteria that may help answer these questions. Acidocalcisomes are found in archaea, protista, bacteria, animalia, plantae and fungi [117,118] and this universality suggests that their origin predates any “Last Universal Common Ancestor” and that they are one of the absolute essentials for the emergence of cellular life [14]. This conjecture is supported by the finding that a 57 amino acid segment of the acidocalcisome protein PF03030, a V-H + PPase, is almost completely conserved across all branches of life [118]. Moreover, acidocalcisomes reveal the ongoing importance of the molecular complementarity between the SUMIs insofar as they are rich in PolyP and calcium and have interactions with polyamines (see above).

In our hypothesis of prebiotic evolution, the composome population progressively became enriched in non-equilibrium composomes in which the interactions between the constituents were weaker and more dynamic than in equilibrium composomes. These relatively fragile composomes may have been kept together by the forces generated by an early form of coupled transcription-translation in which the nascent products interacted with extensive structures such as a membrane. Is there evidence for such forces and for roles for the SUMIs in the modern descendants of non-equilibrium hyperstructures?

The non-equilibrium type of hyperstructure is exemplified by the nucleolus in eukaryotic cells and, by “nucleolus-like” microcompartments in prokaryotic cells where rRNA genes are transcribed and where ribosomal proteins assemble onto the nascent rRNA, so bringing together genes encoding rRNA and rproteins, nascent RNA, as well as nascent ribosomal proteins and their genes [119]. A non-equilibrium structure requires a flow of energy and this requirement would correspond to the appearance of the putative ribosomal hyperstructure in *E. coli* under rapid growth conditions [26,120]. The non-equilibrium composomal ancestor of the ribosomal hyperstructure may also have been the ancestor of the EF-Tu hyperstructure. EF-Tu is a GTPase that delivers amino-acylated tRNAs to the ribosome during the elongation step of translation and that, in bacteria, forms filaments and networks [121,122] which may have a role in the sensing of metabolic activity [123]. Coupling transcription and translation to insertion into the membrane (transertion) or to assembly into ribosomes can expand or condense, respectively, the bacterial nucleoid [120,124]. Such transertion can generate forces sufficient to resist turgor pressure [125]; significantly, therefore, truncating EF-Tu results in cell lysis [126].

In the scenario of the prebiotic ecology, the organization of SUMIs into equilibrium composomes, such as acidocalcisomes, and into non-equilibrium composomes, such as an “EF-Tu/ribosomal” composome (or indeed composomal precursors of any of the modern coupled transcription-translation hyperstructures), may have involved a form of peptide synthesis that preceded polynucleotide-directed template synthesis. In modern cells, spermine stabilizes the conformation of the yeast Tyr-tRNA and Phe-tRNA, stimulates their binding to the A and P sites of the *E. coli* ribosomes [127], whilst in other studies polyamines have been shown to act at both the initiation and elongation steps in translation, and to affect the levels of proteins encoded by the polyamine modulon such as the RNA polymerase sigma subunit, RpoS, and Fis, which itself increases the level of rRNA and tRNA [20]. Covalent modification by PHB would have facilitated the interactions between peptides, membranes and nucleic acids within the composomal ancestor of the ribosomal and EF-Tu hyperstructures given that in *E. coli*, ribosomal/EF-Tu hyperstructure constituents that undergo modification by PHB include the elongation factors EF-Tu and EF-Ts themselves, ribosomal proteins RplL and S1, and RNA polymerase subunit, RpoA [17]. PolyP has been shown to catalyze peptide synthesis in a variety of aqueous prebiotic conditions [128-130] aided by magnesium as a catalyst [131]. More importantly, phosphate binding on proteins often involves a very simple, highly conserved motif of three to six residues [132,133], suggesting that PolyP might have provided a template for the synthesis of the peptides to which it binds. This has led to the suggestion that a PolyP-peptide synergy linking phosphate metabolism with peptide synthesis was one of the key steps in the emergence of life [134]. One of the results of this linkage could have been the PolyP-template-directed synthesis of the peptide precursors of the modern translational machinery and the various kinases, phosphatases and ion channels that are incorporated into acidocalcisomes. A similar mechanism linking calcium-directed templating of PolyP-stimulated peptide synthesis could have produced the highly conserved modules, such as EF hands, made up of the dozen amino acids that form most calcium binding sites in modern proteins [135]. In this way, a localized primitive metabolism could have evolved in the absence of cellular life and provided one of the key components incorporated into it.

## Testing the hypothesis

SUMIs are essential components of all living systems and their fundamental properties and interactions must therefore be one of the key foci of origins of life research. Particular attention should be paid to compositional aggregates of SUMIs such as acidocalcisomes. The roles we suggest that SUMIs played in the organization of life facilitate several experimental predictions. First, SUMIs should themselves self-organize into simple compositional aggregates, a prediction that is easily testable. Second, adding SUMIs such as PolyP and calcium to Urey-Miller type experiments [136] should have a dramatic effect on the nature of the resulting products. The addition of one SUMI should influence the appearance of the SUMIs with which it interacts. Seeding the starting mix with the SUMIs should lead to the formation of self-aggregating complexes and produce specific peptide sequences that can interact functionally with phosphates, PHB, polyamines, etc. In such modified reruns of the Urey-Miller experiment, it would be important to add a step to counter-select those molecules that do not interact with one another and that therefore might be preferentially eliminated by exposure to *uv* radiation; we predict that, by going through many cycles, the wide variety of molecules generated in the original Urey-Miller experiment would be narrowed onto the limited number of those that interact due to molecular complementary. Third, addition of SUMIs should foster the production of components required by experiments to construct minimal cells from phospholipid components [137]. The testable implications also include the existence of complementarity between many of the hundreds of proteins that have evolved to regulate and make use of SUMIs in modern cells [22].

## Implications of the hypothesis

Attributing a major role to SUMIs in the origins of life has several advantages. First, they could have provided *sources* of carbon, nitrogen and phosphate that were readily useable by protocells. If, like PolyP or lipids, they were already there before cells appeared, or if, like PHB and polyamines, they could have readily been made by the first cells, it would be surprising if these cells had eschewed them early in evolution only to exploit them later.

Second, SUMIs could have acted as *stores* of carbon, nitrogen and phosphate that allowed protocells to benefit from periods of excess or unbalanced supply of these necessities; such stores allow many modern cells to survive stresses such as nutrient deprivation and could have allowed protocells to survive them too.

Third, SUMIs have *particular advantages* for cells, both separately and in combination, that make them leading candidates for having been the major constituents of protocells. PolyP is a potent source of energy. PHB can facilitate the interaction of proteins with one another and with nucleic acids. PolyP and PHB assemble together in lipid membranes to form ion channels and pumps the specificity of which can be modified by association with proteins [44] and hence, in the prebiotic ecology, by association with peptides. Polyamines can alter the distribution and conformation of DNA [54,55]; they can also strengthen membranes, alter lateral and transverse distributions of lipids, and promote membrane fusion. Such membrane properties are fundamental to certain models of life in which evolution occurs via the fission-fusion of membraneous composomes [11], in which polymerisation occurs at the interface between membrane domains [10] and in which the concentrating of reactants occurs via encapsulation [66].

Fourth, the *interactions* between SUMIs could have limited the otherwise enormous space of phenotypes that would have been available to protocells in the prebiotic ecology; in this scenario, a great diversity of abiotic molecules existed in protocells, possibly in a huge number of combinations *alias* phenotypes; the problem here is how to generate a few coherent phenotypes on which natural selection can act to permit evolution. Modern cells face the same problem and how they solve it is uncertain. One of the numerous possibilities is that molecular complementarity played a fundamental role by favoring the accumulation of those molecules that were stabilized by interactions with other molecules (and indeed with themselves) [4]. The SUMIs satisfy the requirements of molecular complementarity since there are interactions between PolyP and PHB, between lipids, PolyP and PHB, between polyamines and lipids, between PolyP and polyamines, and between PHB, oligonucleotides and peptides (although we only mention them here, note too reports of interactions between polysaccharides and nucleic acids [138] and between polysaccharides and amino acids [139]). Some of these interactions, like those between polyamines and lipids result in increased stability. Another possibility proposed to limit phenotype space in modern cells is that of the existence of a level of organization intermediate between the macromolecule (or gene) and the cell: this would be the level of assemblies of macromolecules that perform a function, *alias* hyperstructures [3]. SUMIs can assemble into hyperstructures as in the case of PolyP granules or acidocalcisomes [14], of granules of high molecular mass PHB [19], and of polyamines interacting with nucleic acids or with proteoglycans [140]. Such hyperstructures may be the descendants of the composomes in which these molecules were associated and perpetuated.

Evidence that SUMIs lie at the root of the evolution of cellular coordination can be drawn from the number of regulatory processes dependent on them in modern cells. Polyamines up-regulate approximately 300 species of mRNAs through polyamine stimulation of the synthesis of various transcription factors such as Cya, RpoS, FecI, and Fis at the level of translation [20], PolyP affects (via *ppk1*) around seven hundred genes in *P. aeruginosa* [14], PHB affects, via covalent modification, well over a hundred proteins in *E. coli* [17]. It is important to note here that some of these effects are due to the fact that SUMIs interact with, or indeed constitute (in the case of lipids), some of the cell’s largest structures. Such structures as membranes or chromosomes are major regulators in their own right. The membrane acts as global regulator via the dynamics of membrane domains [67] whilst the chromosome acts as a global regulator via a gradient of supercoiling [141]. In this respect, it is easy to see how cells might have evolved to allow calcium to affect the expression of over a hundred genes in *E. coli* [110]: even without calcium-binding proteins in protocells, condensation and decondensation of calcium onto and from DNA would have altered its properties [93]. Perhaps not surprisingly, in modern *E. coli*, calcium modulates the activity of DNA gyrase, the enzyme responsible for increasing supercoiling [111].

In this context of global regulation, it is tempting to trace the origins of one of the major regulatory systems in modern cells, protein phosphorylation, to the SUMIs. Within a composome, the interaction of PolyP with peptides could have provided them with energy for catalysis; such interaction would have been facilitated by the presence of PHB in the composome; such presence would itself be facilitated if the peptides themselves had undergone covalent modification by PHB at multiple residues; such modification could be prevented or even facilitated by modification of these residues by a phosphate donated by PolyP. Hence an elaborate dance of SUMIs within composomes may have simultaneously produced enzymic catalysis and regulation.

Fifth, SUMIs could have played the key role in *life on the scales of equilibria* [6]. Many modern cells have to reconcile the conflicting constraints of growing fast in favorable conditions and resisting stresses in harsh one. It has been argued that life for these cells consists in generating phenotypes that allow populations of cells to maximize their chances of avoiding extinction by going into – and moving between – the two large attractors of growth and survival [6]. In this hypothesis, such “life on the scales” results from the dynamics of two large types of hyperstructures, equilibrium and non-equilibrium. Assuming that a similar selection determined the fate of protocells, the question arises whether hyperstructures comprising SUMIs (for example, granules of PHB and of PolyP) have properties that would allow them to both regulate and respond to transitions between the survival and growth attractors. It turns out that they do have these properties, at least in modern cells. For example, in the case of escaping from an attractor, PolyP can go between cytosolic and granular forms in *C. glutamicum* [37] and PolyP bodies are believed to allow metabolism in *A. ovalisporum* to restart after dormancy [36]. How might SUMI hyperstructures change to allow transitions in life styles? Calcium condensation onto – and decondensation from – hyperstructures might play a key role here [6]; such condensation, which can lead to compaction and decompaction of charged polymers such as DNA [93], is temperature-dependent with ions condensing onto charged linear polymers at higher temperatures and decondensing at lower ones. It is therefore intriguing that *E. coli* L-forms, which may manifest some of the characteristics of protocells, can grow optimally in 0.1 mM calcium at 32°C and in 1.0 mM calcium at 37°C [142].

## Reviews

### Reviewer 1 (Doron Lancet)

“This paper is revealing and important, but requires a major overhaul of the presentation flow.

The major idea, as far as I reckon, is that that there are substances, playing key roles in present-day living cells, which are more likely to have been early “jump-starters” for life as compared to the usually implicated “mainstream” compounds – DNA, RNA and proteins. This idea is appealing, because in the eyes of many, the latter three complex, sequence-centered biopolymers are rather unlikely to have emerged abiotically under early prebiotic conditions.

Three of the potentially prebiotic compounds innovatively implicated by the authors are polymers/oligomers. The first is polyphosphate, chains of diester-bonded orthophosphate groups (PolyP), the second is a homopolymeric polyester, polyhydroxybutyrate (PHB), and the third is a class of relatively short oligomers – polyamines. A fourth implicated component is lipids and a fifth is divalent calcium ions. The authors coin the acronym SUMIs for such compounds – simple universal molecules and ions. The name is not very appropriate, as there is not much that is universal about such compounds, and there is little in common between the polymers lipids and ions, so it would be good to change the acronym. I do admit that PolyP, PHB and polyamines (heretofore called PPP) are indeed simple, in being homopolymeric (nearly so for polyamines), and not portraying the combinatorial complexity of sequence-based heteropolymers. Lipids are a class of their own in this respect, being essentially monomeric, but share the attribute of lacking sequence-related combinatorics.

Two special attributes of PPP emerge from the admirable literature search presented in this paper: 1) They are ubiquitously seen in living cells (with special emphasis on bacteria), and found to participate in numerous contemporary cell functions; 2) They show a curious capacity to interact non-covalently (sometime covalently) with each other and with other components (e.g. proteins) and form supramolecular assemblies. Consequently, the most fundamental hypothesis of the paper, in my view, is that compounds such as PPP and lipids, with the mediation of metal ions (first and foremost calcium), may form readily under prebiotic/abiotic conditions. They are endowed with a capacity, evidenced in contemporary organisms, to form noncovalent complexes that have biochemical functions surprisingly similar to those of their much more elaborate, sequence-based descendants – proteins and RNA. They thus are natural candidates for a transition from a “primordial soup” to full-fledged living cells. I strongly suggest that this clear line of argument become the spine of a revised paper.

Here are points of criticism and suggestions for improvement:

1. The abstract requires a major revision. Its first paragraph begins with the most obscure sentence in the entire paper: “The solutions to these problems are likely to be found with the organic and inorganic molecules and inorganic ions that constituted the first cells …”. Which problems? In what way are the mentioned substances a solution? Similarly, the last sentence of the first paragraph: “Here, we explore the possibility that the direct interactions between PHB, PolyP, polyamines and lipids - modulated by calcium - played a central role in solving the fundamental problems faced by early and modern cells”. Which fundamental problems? The second paragraph does not make the readers’ life easier: “We reason that since these SUMIs are important for modern cells and were probably abundant and available for the first cells, there may be evidence for their interactions in modern cells.” If the authors are trying to decipher protocellular function, why does the punchline address modern cells? The last abstract paragraph is just as confusing: terms like “prebiotic ecology” and “initial phenotype space” are not too revealing and should be better left out.
2. Some of the confusing nature of the latter part of the abstract is reflected in the third point of the “Presentation of the hypothesis” section: “(3) these interactions constituted a system of global regulation that limited phenotype space to the two large attractors of growth and survival and to the transitions between them”. These obscure terms and seemingly formal arguments are never defined or supported in the paper, and should better be left out completely.
3. The paper is considerably disorganized. The aims and hypotheses are scattered throughout and are quite difficult to fathom. It is essential that a clear and concise presentation of the hypothesis is provided in the abstract and in the hypothesis section. I suggest to lump all background information, currently found in the Background chapter as well as in chapters specifically named for the PPP compounds, lipids and calcium into one portrayal, that will summarize all previously published results and observations before presenting the hypothesis. The hypothesis section should then be devoted purely to presenting all the authors’ conjectures, based on all the information displayed beforehand. To exemplify this point, in the current hypothesis section does not include a hypothesis point shown 2 paragraphs earlier in the Background section - …source and a reservoir of carbon, phosphorus and nitrogen…; In turn the current hypothesis section has a point not obviously belonging there “roles…can still be discerned in modern cells”.
4. Non-covalent assemblies are often referred to in the paper as “composomes”. Indeed composomes (REF 5) have been invoked as non-covalent heterogeneous molecular assemblies present at the early stages of Life’s emergence. But the most crucial aspect of composomes is that they are dynamic structures that manifest rudimentary information transfer from one growth-split generation to another. It would be good to address this latter property in reference to the molecular non-covalent complexes invoked in the present paper. This is particularly important in relation two of the hypothesis points: “the interactions between SUMIs determined the behavior of composomes”. It is really necessary to back such a statement with specific relationships.
5. Prebiotic non-covalent structures, composomes, hyperstructures, present day organelles (e.g. acidocalcisome) and conjectural assembles such as EF-Tu/ribosomal structures are all intermixed in the paper’s argumentation, particularly in the From composome to hyperstructure section, in ways that require considerable further clarification. What happened first? What was the evolutionary progression? What were the driving forces for such? In fact the abovementioned section contains many statements that should be part of a well-balanced hypothesis section (see comment 3). The final sentence of the second paragraph in this section addresses energy considerations not at all mentioned in the hypothesis section – needs to be corrected. The text within this very sentence “…via integrative interactions between hyperstructures, convert this energy into biologically useful forms” is truly obscure and unless supported by more formal arguments needs to be eliminated.
6. In the Testing the hypothesis section, the idea of adding to compounds highlighted by this paper to a Miller-Urey type experiment makes little sense. The experiment addresses the production of organic compounds from inorganic gases. It produces a plethora of small and large molecules that were never fully analyzed. The experiment has no delineation of a progression from the organic compounds to the next steps in prebiotic evolution. So even if the authors meant adding compounds such as PolyP and PHB to the organic residue of the Miller Urey experiment, this involves no testable predictions. On the other hand, it is quite clear that experiments should be suggested to investigate the abiogenesis of PPP.

Minor points:

7) The authors strive to portray events that preceded true cellular life, but often inappropriately use the term “first cells”, likely intending to say something like “protocells”.
8) I do not see the validity of the statement in the Interactions with Calcium section: “The first cells would have had to cope with huge amounts of extracellular calcium and it is therefore not surprising that calcium is involved in many different processes…”. There were large amounts of many ions, and a role for an ion is likely brought about by evolutionary emergence of molecular mechanisms and not by need “to cope”.

### Reviewer 2 (Kepa Ruiz-Mirazo)

#### Summary

This article proposes that there is a set of simple, universal molecules and ions (so called ‘SUMIs’ by the authors) that played a fundamental role in the origin and first evolution of living cells. To support that claim, the scientific literature is extensively reviewed, highlighting the multiple interactions and diverse functions realized by this type of molecules (polyphosphates, polyhydroxybutyrate, polyamines, lipids and calcium, in particular) in extant cells (e.g., *E. coli*).

#### Critical assessment

The paper is correctly written (good standard of English) and well supported in previous work. Well, perhaps I do miss some references to Robert Shapiro and Christian de Duve, who defended similar hypotheses (‘oligomer worlds’) for life’s origins in the past. Personally, I feel very sympathetic with the approach, because my own line of work in this field has tried to be rigorous in its bottom-up assumptions, which means starting really simple (molecularly speaking). Biopolymers (like RNA, DNA and proteins) should eventually come into the picture, of course, but the most reasonable position is to conceive of them as rather “latecomers” in any sequence of prebiotic transitions (like the authors of this paper do).

As far as the hypothesis goes, we should distinguish between the general claim that simple organic molecules ought to be used for starters in origins of life research, which is fine but not so original, or the general mechanisms proposed to drive those first stages (e.g., ‘molecular complementarity’, ‘compositional inheritance’, formation of ‘hyperstructures’), which I do not find so informative or explanatory, really, and the specific suggestion given (on polyP, PHB, polyamines, etc.) which may hold some interest and could be worth exploring.

Unfortunately, the paper does not provide any direct evidence of the prebiotic relevance or role that those components could have. It is just a bold hypothesis, based on a thorough exercise of ‘molecular paleontology’, to be tested in the future -- if someone finds it attractive enough.

#### Minor points and suggestions

In the pdf provided, the ‘background’ subsection is missing from the abstract.

Nowhere is a definition of ‘molecular complementarity’ given in the text: there are many types of non-covalent interactions that hold things together in biological systems. A strategy of grouping all of them under the same term does not seem very helpful.

If possible, provide a better example (than the nucleolus or EF-Tu) for a non-equilibrium hyperstructure that could be of prebiotic significance”.

### Response to Doron Lancet

The authors coin the acronym SUMIs for such compounds – simple universal molecules and ions. The name is not very appropriate, as there is not much that is universal about such compounds, and there is little in common between the polymers lipids and ions, so it would be good to change the acronym.

We are not sure why the reviewer thinks that “there is not much universal” about lipids and these polymers. Lipids, PHB and PolyP are, to our knowledge, present in all species whilst calcium is everywhere (the problem is often keeping it out). That said, polyamines have been reported as absent in Staphlococcus aureus and we now cite this paper. The suggestion for a different acronym is a good one but we would prefer to stick with the original one (which has a meaning in Japanese – a sumi is a stick of ink used by painters – that is somewhat appropriate).

1) The abstract requires a major revision. Its first paragraph begins with the most obscure sentence in the entire paper: “The solutions to these problems are likely to be found with the organic and inorganic molecules and inorganic ions that constituted the first cells …”. Which problems? In what way are the mentioned substances a solution? Similarly, the last sentence of the first paragraph: “Here, we explore the possibility that the direct interactions between PHB, PolyP, polyamines and lipids - modulated by calcium - played a central role in solving the fundamental problems faced by early and modern cells”. Which fundamental problems?

Unfortunately, a cut-and-paste error led to the absence of the required information in what should have been the background. We have now inserted this: “Fundamental problems faced by the precurors of cells (protocells) and by their modern descendants include how to go from one phenotypic state to another, how to escape from a basin of attraction in the space of phenotypes, how to reconcile conflicting growth and survival strategies (and thereby live on ‘the scales of equilibria’) and, more generally still, how to create a coherent, reproducible phenotype at all from a multitude of constituents”.

The second paragraph does not make the readers’ life easier: “We reason that since these SUMIs are important for modern cells and were probably abundant and available for the first cells, there may be evidence for their interactions in modern cells.” If the authors are trying to decipher protocellular function, why does the punchline address modern cells?

We have rewritten this section and have incorporated much of the paragraph proposed by the reviewer: “We review the evidence that the SUMIs (1) were probably abundant and available for the first cells, (2) are widespread in modern cells, (3) interact with one another and with other constituents in modern cells to create new structures with new functions that are surprisingly similar to those of proteins and RNA, and (4) are essential to helping modern bacteria create coherent phenotypes. We reason therefore that the SUMIs are natural candidates for both reducing the immensity of phenotype space and making the transition from a “primordial soup” to full-fledged living cells”.

The last abstract paragraph is just as confusing: terms like “prebiotic ecology” and “initial phenotype space” are not too revealing and should be better left out.

We have rewritten this paragraph, we define composomes and now say in this paragraph: “prebiotic ecology of co-evolving populations of composomes” and “initial phenotype space of composomes”. Later in the text, we try to make it clear why these concepts are essential to our hypothesis (see below).

“(3) these interactions constituted a system of global regulation that limited phenotype space to the two large attractors of growth and survival and to the transitions between them”. These obscure terms and seemingly formal arguments are never defined or supported in the paper, and should better be left out completely.

We have put these problems upfront in our paper and, rather than delete them, we have tried to explain them better. We say in the Background: “The first of these fundamental problems is how to generate reproducible, coherent phenotypes from a large number of effectively different molecules. In modern cells, this number runs into thousands - if not millions –if genes and other nucleic acid sequences are considered as separate entities, if post-translational modifications are taken into account and if the small molecules of metabolism are included. The combination of the activities of these molecules generates the phenotype on which natural selection acts. Since there would appear to be an almost unlimited number of such combinations, there should be an almost unlimited number of phenotypes. … The upshot of all this is that probably less than a hundred different hyperstructures, created in part by molecular complementarity, determine the phenotype of the bacterium. This means that the number of hyperstructures is orders of magnitude less than the number of macromolecules so the number of phenotypes resulting from combinations of hyperstructures is much smaller than the number resulting from combinations of macromolecules. In other words, phenotype space is dramatically reduced but remains huge. … A second, fundamental problem is how to generate phenotypes that can satisfy the incompatible requirements of survival and growth. This is the problem of ‘Life on the scales’ whereby cells are damned if they simply grow (since they risk being destroyed if conditions turn bad) and damned if they eschew growth, for example, to sporulate (since they risk being out-competed by other, growing, cells if conditions remain good) {Norris, 2012 #5109}. To simplify it, at one extreme, survival requires a cell that approaches an equilibrium state in which it is relatively static and robust but does not grow (with interactions between cellular constituents are strong and stable) whilst, at the other extreme, growth requires a cell in a non-equilibrium state in which it is highly dynamic and metabolically active but risks death (with interactions between cellular constituents that are weak and unstable). Moreover, cells must be able to go between these states. One of the solutions adopted by modern bacteria is to have a cell cycle that gives daughter cells with different phenotypes as evidenced by the division of Caulobacter crescentus into stalked and swarmer cells each of which can generate the other {Bowman, 2013 #5758}. In the hyperstructure hypothesis, these different phenotypes are conferred by different combinations of equilibrium (technically, quasi-equilibrium) and non-equilibrium hyperstructures {Norris, 2007 #2798}”.

I suggest to lump all background information … before presenting the hypothesis. The hypothesis section should then be devoted purely to presenting all the authors’ conjectures, based on all the information displayed beforehand. To exemplify this point, in the current hypothesis section does not include a hypothesis point shown 2 paragraphs earlier in the Background section - …source and a reservoir of carbon, phosphorus and nitrogen…; In turn the current hypothesis section has a point not obviously belonging there “roles…can still be discerned in modern cells”.

In the limit, there are two ways to present a hypothesis. One is the way proposed by the reviewer. The disadvantage is that the reason for the choice of facts that precede and build up to the hypothesis is unclear until the reader reaches the hypothesis. Of course, this is fine for a review in which the hypothesis is only of minor importance. The other way, which we prefer and which is recommended by the journal, is to spell out the hypothesis first. This might seem to have the disadvantage that at least some background information still has to be given first. However, we subscribe to the view that this background should be about the nature of “what is the problem?” rather than about the properties of PolyP, PHB, polyamines, lipids and calcium, so we would prefer to retain our original structure. That said, the reviewer’s suggestion has several merits and we have therefore included much more information about the SUMIs in the Background section.

We have rewritten the presentation of the hypothesis and have added to it the missing bit about the SUMIs being a reservoir of carbon, phosphorus and nitrogen. The reviewer says that the statement that “roles … (of the SUMIs)…can still be discerned in modern cells” is out of place in the presentation of the hypothesis. As we see it, this statement is an assumption (i.e., a hypothesis in its own right) that justifies our gathering information in support of our principal hypothesis and its best place is therefore in the presentation section. We have, however, modified the phrase by adding “in their universal utilization of ribosome microcompartments and acidocalcisomes to regulate key cellular processes”.

Non-covalent assemblies are often referred to in the paper as “composomes”. Indeed composomes (REF 5) have been invoked as non-covalent heterogeneous molecular assemblies present at the early stages of Life’s emergence. But the most crucial aspect of composomes is that they are dynamic structures that manifest rudimentary information transfer from one growth-split generation to another. It would be good to address this latter property in reference to the molecular non-covalent complexes invoked in the present paper.

This is particularly important in relation two of the hypothesis points: “the interactions between SUMIs determined the behavior of composomes”. It is really necessary to back such a statement with specific relationships.

The problem here is that the reviewer and his group have coined an excellent term which we should not and cannot avoid! Composomes, in our version of the concept, fall into two classes, non-equilibrium and (quasi-)equilibrium, the former being much more dynamic than the latter (which we previously termed ‘protocells’). This is important because the behaviours of the two classes of composomes would have been very different: one class is equipped for growth and the other for survival; moreover, we believe that the phenotype of modern cells is governed by non-equilibrium and equilibrium classes of the modern counterparts of composomes (that is, hyperstructures). There is sufficient evidence out there to implicate the SUMIs in growth, survival and the transitions between these behaviours. We therefore now say in the Background:

Although the current scenario of the prebiotic ecology offers solutions to fundamental problems, these solutions are incomplete. How was phenotype space sufficiently constrained by composomes to have enabled natural selection to act effectively? How did composomes or collections of composomes go from an equilibrium state to a non-equilibrium one and back again? How exactly was energy generated and catalysis achieved?Addressing these questions by invoking peptides and oligonucleotides would be understandable. There are, however, other molecules that merit consideration. Not so long ago, the late Arthur Kornberg chided the scientific community for dismissing polyphosphate (PolyP) and its metabolism as a mere “molecular fossil” {Kornberg, 2008 #5480}. In modern cells, PolyP is implicated in quorum sensing, biofilm formation, motility, virulence, sporulation phosphate storage and energy metabolism {Rao, 2009 #3546}. PolyP is not alone in receiving insufficient attention. Short chain poly-(R)-3-hydroxybutyrate (PHB) can form ion and DNA uptake channels (with PolyP) and pumps {Das, 1997 #983} {Reusch, 1997 #2028} and may directly modulate interactions between proteins, nucleic acids and membranes {Huang, 1996 #807} {Reusch, 2002 #1615}; moreover, PHB can act as a carbon store {Wahl, 2012 #5157}. Polyamines bind to nucleic acids and proteins, decrease membrane permeability, may help cells survive abiotic stresses {Igarashi, 2006 #2726} {Groppa, 2008 #2724}; polyamines may also act as nitrogen stores.

We also now say in the section “From composome to hyperstructure (and back again)”:

At a very early stage of the evolution of the prebiotic ecology, equilibrium composomes could have exploited the free energy associated with the attraction of peptides to the boundaries of lipid domains {Netz, 1996 #818} to drive polymerization. Such boundaries can concentrate, align and orientate monomers, reduce hydrolysis and increase condensation. Indeed, a model of abiotic catalysis shows the free energy change from the redistribution of peptides within domains is sufficient to drive the formation of the peptide bond; the resulting polymerization is significant and the equilibrium distribution of polymer lengths is shifted towards longer peptides. This model is supported by the observation of shifts in equilibria in reactions in organic solvents {Deschrevel, 1992 #1595}{Hitz, 2000 #6148} and by the 1011 increase in reaction rates due to orientation effects {Milstien, 1970 #1594}. etc.

Prebiotic non-covalent structures, composomes, hyperstructures, present day organelles (e.g. acidocalcisome) and conjectural assembles such as EF-Tu/ribosomal structures are all intermixed in the paper’s argumentation, particularly in the From composome to hyperstructure section, in ways that require considerable further clarification. What happened first? What was the evolutionary progression? What were the driving forces for such? In fact the abovementioned section contains many statements that should be part of a well-balanced hypothesis section (see comment 3). The final sentence of the second paragraph in this section addresses energy considerations not at all mentioned in the hypothesis section – needs to be corrected. The text within this very sentence “…via integrative interactions between hyperstructures, convert this energy into biologically useful forms” is truly obscure and unless supported by more formal arguments needs to be eliminated.

We have rewritten the “From composome to hyperstructure” section to make it clear what happened first:

“In our hypothesis, boundaries or interfaces between combinations of SUMIs and other molecules abounded in the equilibrium composomes which dominated early prebiotic evolution. Catalysis on these interfaces resulted from the dynamic effects on the composomes of changes in the physical and chemical nature of their environment, and division to generate a network on interacting polymers. This process was guided by the molecular complementarity between the SUMIs and between the monomers and polymers with the SUMIs”.

We have transferred hypotheses to the hypothesis section:

“The equivalents of the non-equilibrium types of composome in which macromolecules are synthesized are epitomized by the nucleolus in eukaryotic cells and by the “nucleolus-like” hyperstructures in prokaryotic cells as well as by the other prokaryotic hyperstructures in which transcription and translation are coupled. The equivalents of the equilibrium types of hyperstructures are epitomized by acidocalcisomes which help regulate osmolarity and pH and which are found in every major branch of life”.

We have changed “The resulting composomes were able to accumulate phosphates while regulating calcium and other ion concentrations and fluxes in order to store the resulting energy and, via integrative interactions between hyperstructures, convert this energy into biologically useful forms” into “Could the molecular complementarity between SUMIs involved in acidocalcisome metabolism have resulted in the formation of self-organizing composomes in which integrative interactions between SUMIs (in the appropriate conditions) converted energy into biologically useful forms such as polymerization? Universality and conservation are criteria that may help answer these questions. etc.”

In the Testing the hypothesis section, the idea of adding to compounds highlighted by this paper to a Miller-Urey type experiment makes little sense. The experiment addresses the production of organic compounds from inorganic gases. It produces a plethora of small and large molecules that were never fully analyzed. The experiment has no delineation of a progression from the organic compounds to the next steps in prebiotic evolution. So even if the authors meant adding compounds such as PolyP and PHB to the organic residue of the Miller Urey experiment, this involves no testable predictions. On the other hand, it is quite clear that experiments should be suggested to investigate the abiogenesis of PPP.

We have modified the Testing section to clarify the experiment we mean and to respond to the point about abiogenesis of the SUMIs: “Second, adding SUMIs such as PolyP and calcium to Urey-Miller type experiments {Miller, 1959 #5276} should have a dramatic effect on the nature of the resulting products. The addition of one SUMI should influence the appearance of the SUMIs with which it interacts. Seeding the starting mix with the SUMIs should lead to the formation of self-aggregating complexes and produce specific peptide sequences that can interact functionally with phosphates, PHB, polyamines, etc. In such modified reruns of the Urey-Miller experiment, it would be important to add a step to counter-select those molecules that do not interact with one another and that therefore might be preferentially eliminated by exposure to uv radiation; we predict that, by going through many cycles, the wide variety of molecules generated in the original Urey-Miller experiment would be narrowed onto the limited number of those that interact due to molecular complementary”.

The authors strive to portray events that preceded true cellular life, but often inappropriately use the term “first cells”, likely intending to say something like “protocells”.

Yes. We have now replaced “first cells” and “early cells” with “protocells”.

I do not see the validity of the statement in the Interactions with Calcium section: “The first cells would have had to cope with huge amounts of extracellular calcium and it is therefore not surprising that calcium is involved in many different processes…”. There were large amounts of many ions, and a role for an ion is likely brought about by evolutionary emergence of molecular mechanisms and not by need “to cope”.

Calcium would initially have been a problem for protocells. We now say: “Protocells would have had to cope with problems such as the solubility of phosphate posed by huge amounts of extracellular calcium and it may be that, in overcoming these problems, calcium became involved in many different processes as evidenced in modern bacteria”

### Response to Kepa Ruiz-Mirazo

#### Critical assessment

The paper is correctly written (good standard of English) and well supported in previous work. Well perhaps I do miss some references to Robert Shapiro and Christian de Duve who defended similar hypotheses (‘oligomer worlds’) for life’s origins in the past. Personally I feel very sympathetic with the approach because my own line of work in this field has tried to be rigorous in its bottom-up assumptions which means starting really simple (molecularly speaking). Biopolymers (like RNA DNA and proteins) should eventually come into the picture of course but the most reasonable position is to conceive of them as rather “latecomers” in any sequence of prebiotic transitions (like the authors of this paper do).

We now cite both Shapiro and de Duve.

As far as the hypothesis goes we should distinguish between the general claim that simple organic molecules ought to be used for starters in origins of life research which is fine but not so original or the general mechanisms proposed to drive those first stages (e.g. ‘molecular complementarity’, ‘compositional inheritance’, formation of ‘hyperstructures’), which I do not find so informative or explanatory really and the specific suggestion given (on polyP, PHB, polyamines etc.) which may hold some interest and could be worth exploring. Unfortunately the paper does not provide any direct evidence of the prebiotic relevance or role that those components could have.

We now mention in the Abstract the role that these components could have had.

It is just a bold hypothesis based on a thorough exercise of ‘molecular paleontology’, to be tested in the future -- if someone finds it attractive enough.

#### Minor points and suggestions

In the pdf provided the ‘background’ subsection is missing from the abstract.

We have now added it.

Nowhere is a definition of ‘molecular complementarity’ given in the text: there are many types of non-covalent interactions that hold things together in biological systems. A strategy of grouping all of them under the same term does not seem very helpful.

We now say “Molecular complementarity occurs when the shapes of molecules or macromolecules fit one another such that physical, non-covalent interactions result in their associating reversibly with one another {Root-Bernstein, 1997 #2554} {Shapiro, 2006 #6137}. Molecular complementarity underlies functions such as information storage and translation, enzymatic reactions, structural self-organization and protection of molecules from degradative processes”. and “In this scenario, the solution again lay in molecular assemblies and molecular complementarity: molecules were abiotically created and destroyed but a subset of molecules was preserved because their complementarity led to associations between them that protected them from degradation; these interacting molecules then accumulated in the form of molecular and macromolecular assemblies or composomes – the ancestors of modern hyperstructures – which possessed new properties and which exhibited compositional inheritance”.

If possible provide a better example (than the nucleolus or EF-Tu) for a non-equilibrium hyperstructure that could be of prebiotic significance.

We gave the example of the EF-Tu/ribosomal hyperstructure because it is fundamental to RNA and protein synthesis and because we focus on it elsewhere in the text. We have now added the example of the modern, coupled transcription-translation hyperstructures.

## Abbreviations

SUMIs: Simple Universal Molecules and Ions
PolyP: Polyphosphate
PHB: Poly-(R)-3-hydroxybutyrate
(p)ppGpp: Guanosine 5′-triphosphate, 3′-diphosphate and guanosine 5′-diphosphate, 3′-diphosphate
